# High-Specificity Digital Architecture for Real-Time Recognition of Loss of Balance Inducing Fall

**DOI:** 10.3390/s20030769

**Published:** 2020-01-31

**Authors:** Daniela De Venuto, Giovanni Mezzina

**Affiliations:** Department of Electrical and Information Engineering, Politecnico di Bari, 70125 Bari, Italy; daniela.devenuto@poliba.it

**Keywords:** near falls, loss of balance, pre-impact fall detection, activities of daily life, bio-signals, EEG, EMG

## Abstract

Falls are a significant cause of loss of independence, disability and reduced quality of life in people with Parkinson’s disease (PD). Intervening quickly and accurately on the postural instability could strongly reduce the consequences of falls. In this context, the paper proposes and validates a novel architecture for the reliable recognition of losses of balance situations. The proposed system addresses some challenges related to the daily life applicability of near-fall recognition systems: the high specificity and system robustness against the Activities of Daily Life (ADL). In this respect, the proposed algorithm has been tested on five different tasks: walking steps, sudden curves, chair transfers via the timed up and go (TUG) test, balance-challenging obstacle avoidance and slip-induced loss of balance. The system analyzes data from wireless acquisition devices that capture electroencephalography (EEG) and electromyography (EMG) signals. The collected data are sent to two main units: the muscular unit and the cortical one. The first realizes a binary ON/OFF pattern from muscular activity (10 EMGs) and triggers the cortical unit. This latter unit evaluates the rate of variation in the EEG power spectrum density (PSD), considering five bands of interest. The neuromuscular features are then sent to a logical network for the final classification, which distinguishes among falls and ADL. In this preliminary study, we tested the proposed model on 9 healthy subjects (aged 26.3 ± 2.4 years), even if the study on PD patients is under investigation. Experimental validation on healthy subjects showed that the system reacts in 370.62 ± 60.85 ms with a sensitivity of 93.33 ± 5.16%. During the ADL tests the system showed a specificity of 98.91 ± 0.44% in steady walking steps recognition, 99.61 ± 0.66% in sudden curves detection, 98.95 ± 1.27% in contractions related to TUG tests and 98.42 ± 0.90% in the obstacle avoidance protocol.

## 1. Introduction

Recently, freezing of gait (FOG) and falls received increasing recognition as strongly debilitating features of Parkinson’s disease (PD) [1,2]. By contrast with the tremor, which dominates the early stage of PD [2], falls and FOG are most common in advanced PD stages. The two phenomena seem related to each other, according to the study in [2], in which it is shown how sudden FOG can disturb the balance and, thereby, represents a common cause of falls in PD. Epidemiologic prospective studies conducted with 1-year, or 6-months, follow up [1,2,3,4,5,6], showed that the 45–68% of people with PD experience at least one fall per year, with a large portion (50–86%) falling recurrently [3,4,5]. It is not surprising that including the “near falls” this rate increases up to ~90% [6]. In this context, a near fall is a situation in which, despite a loss of balance, the body-ground impact could be avoided by grasping a support [6].

The clinical presentation in [2] shows that in PD patients most falls result from sudden changes in posture (in particular, turning movements of the trunk), rapid changes in the walking tasks (curve, transfers from the bed or the chair, etc.) or because they try to perform more than one activity simultaneously with walking or balancing. 

In this context, the advances in wireless sensors networks, wearable acquisition devices, and new and more reliable digital signal-processing approaches for kinematic and biosignals analysis prompted the scientific community to develop technological solutions for early fall detection (FD).

The systematic review of FD solutions in [7] showed that body-worn accelerometers can be used to detect impacts and changes in orientation associated with falls. In the same context, the authors in [7] conclude that the accuracy of these FD systems may be improved by jointly using multiple sensors, e.g., signals from smartphone gyroscopes or barometers to define the height changes associated with falls [7,8,9]. These technologies aim to provide fast detection of falls but, at the moment, they are still not able to fully prevent injuries resulting from falls (e.g., hip fractures and traumatic brain injury) [8,9]. For this purpose, the focus of research contextually moved on fall risk assessment. This area of interest oversees the identification of the people’s risk of falling, facilitating in this way early interventions via FD systems. Currently, fall risk-assessment procedures take into account the clinical evaluation of multiple domains such as balance control, mobility, physiology (strength, vision), psychology (fear of falling), cognition and environmental risk [7]. 

In this context, detecting near-falls (or recoverable imbalances) provides new opportunities to identify people with a high risk of falling before an actual fall occurs [10]. Near falls are defined as loss of balance that does not result in a fall because corrective action is taken to recover balance. They typically consist of slips, trips, and missteps. Moreover, since older people who frequently experience near falls are at increased risk of future falls [7,10], remote monitoring of these events during daily life could provide useful information to target falls and related circumstances as part of fall prevention initiatives [7].

Ultimately, an accurate algorithm for the detection of near falls could enhance the quality of existing fall detection systems by reducing false alarms [7].

In this respect, Table 1 summarizes state-of-the-art solutions [11,12,13,14,15] declared to be able to recognize near falls. The table reports the architectures in terms of used acquisition equipment, fall indicators (i.e., the feature(s) to be monitored and classified) and chosen classification method. Table 1 also dedicates a field to the Activities of Daily Life (ADL) and near-fall scenarios included in the discrimination. Finally, the last two rows summarize the declared system performance (i.e., accuracy and efficiency) and the applicability of proposed systems to daily-life and/or ambulatory contexts, as well as their suitability in the context of real-time near falls detection and fall prevention. All the studies selected for the comparison analyze unexpected slippages, classifying them as near falls because all the perturbations analyzed in [11,12,13,14,15] led to balance recovery. 

Table 1 shows that the most used technologies in the loss of balance detection are motion capture systems (MCS) [12,13,15] and inertial measurement units (IMU) [11,14]. Solutions based on MCS are classified as context-aware and typically consists of a set of reflective markers and fixed cameras. For this reason, MCS-based fall detection systems present two limits: they are expensive and only suitable for ambulatory applications [12,13]. Pointing at daily-life applicability, authors in [11,14] propose wearable solutions mostly based on IMU sensors. More in detail, the authors in [11] analyze acceleration and angular velocity from 7 IMU sensors via a machine-learning (ML) approach. In a similar way, the authors in [14] exploit acceleration data from a single device, placed on the waist, to record vertical velocity from trials belonging to the chosen classification clusters (i.e., ADL vs. near falls). In terms of adopted classification methods, the result is that the most used approaches are still based on thresholds, in order to preserve a good speed in system response [12,14,15]. 

Machine-learning based solutions have been also investigated by authors in [11,13]. In this respect, noteworthy is the approach in [13], where the authors use an artificial neural network (ANN) to classify acceleration independent components, providing an interesting tradeoff between overall accuracy and fall-detection timing. 

Table 1 reports the most investigated performance in FD and near-fall detection applications: the fall/imbalance recognition accuracy and the detection time [16]. The system accuracy parameter is composed of the sensitivity and the specificity. For the sake of comparison, all the analyzed works [11,12,13,14,15] share the same sensitivity and specificity definition. The first (i.e., sensitivity, Se (%) in Table 1) is defined as the ratio between the number of successfully detected falls/losses of balance over the total number of recorded perturbations. While, the specificity (Sp (%) in Table 1) is determined by the ratio between the number of successfully detected ADL over the total number of ADL-related trials. Finally, the detection time (DT in Table 1) characterizes the system efficiency. It is defined as the time difference between the fall initiation (that can be uniquely defined) and fall detection. This parameter gives an idea on how rapid the fall-detection system responds to a fall. 

Concerning the applicability field of Table 1, the device’s wearability and the proper specificity characterization relate to the suitability of the application to ordinary-life contexts. While, the detection time under a balance recovery limit (i.e., ~550 ms) [17] determines the system suitability for pre-impact FD strategy improvement.

In this paper, we propose and preliminarily validate a digital architecture for the loss of balance recognition during unexpected slippages potentially inducing fall. The main contributions of the paper concern:

- Fall Indicators. The architecture exploits a novel joint analysis of bio signals: electromyography (EMG) and electroencephalography (EEG).
- High Sensitivity and Specificity. The algorithm robustness is tested both in presence of unexpected slippages (near-fall scenarios) and during four ADL-like tasks: (i) steady walking, (ii) sudden curves, (iii) chair transfers via timed up and go (TUG) test and (iv) balance-challenging obstacle avoidance.
- Quick Loss of Balance Recognition. The system detection time reached by the proposed architecture is conservatively below the maximum intervention time limit for the countermeasures implementation [17].
- Wearability. The proposed architecture is fully based on wireless and wearable sensors, ensuring—together with the high-specificity constraint—the suitability in ordinary life applications.

The architecture proposed here exploits medical evidence from recent studies [18,19,20,21,22,23], according to which the cerebral cortex can regulate the postural stability according to environmental demands [18]. Specifically, the authors in [19,20] proved that low-frequency cortical rhythms (f < 13 Hz) are related to perception and cognitive control. In the loss of balance context, the modulation of the bands θ (4–7 Hz) and α (8–12 Hz) seems to be related to the visual field stabilization and active decoding of data from the vestibular system. Contextually, authors in [21,22,23] concluded that high-frequency cortical rhythms (f > 13 Hz) are commonly related to highly specific motor functions. Specifically, the β bands (i.e., β I, β II, β III), play a main role in muscle firing operations to compensate balance. 

Besides the cortical dynamics’ characterization, the muscular behavior could also be uniquely characterized. In this respect, the authors in [22,23,24,25] demonstrated that for accelerations or decelerations of the supporting surface (e.g., slippage) a low latency response (70–300 ms) occurs in the muscles near the ankles. It results in a muscular pattern characterized by co-contractions between agonist and antagonist muscle bundles [24]. 

Keeping this evidence in mind, the novel architecture exploits electrophysiological measurements from 10 EMG electrodes, to assess the muscular activity, and 13 EEG channels, to analyze the subject’s cortical involvement during reactive response or normal motor planning.

Electrophysiological signals (i.e., EEG/EMG) are synchronously acquired via a central gateway. The gateway streams data to two computational units that distinctly analyze muscular and cortical activity. The unit dedicated to the muscular characterization has two main roles: realizing a binary ON/OFF pattern from muscular activity and triggering the cortical analysis unit. Once triggered, this latter unit quantifies the cortical involvements as the rate of variation in the EEG power spectrum density (PSD), considering the five bands of interest identified by authors in [18,19,20,21,22,23]. The parameters extracted from these units define some neuromuscular features of the subject under monitoring. As a final step, these features are sent to a logical network, which embeds a set of dynamic thresholds from the system calibration phase. In this application, the system calibration progressively builds a conservative range in which the neuromuscular features can be considered as “standard” and, thus, safe for the balance. The expectancy is that the ADL do not strongly affect the cortico-muscular parameters as, instead, happens during a loss of balance. 

The paper is structured as follows. Section 2 outlines the experimental protocols, the setup and the implemented algorithm. Section 3 is dedicated to experimental results. Section 4 proposes a discussion about the system outcomes and Section 5 concludes the paper, presenting future perspectives.

## 2. Materials and Methods

### 2.1. Participants

Nine young and healthy volunteers (8 males, 1 female, 26.3 ± 2.4 years old, 64.5 ± 9.8 kg, 1.71 ± 0.06 m) were enrolled for this study. Six of them contributed to a near-fall scenarios test, while three subjects were actively involved in the system robustness test via ADL-like tasks. Before starting the experimental sessions, all the participants signed the informed consent. Research procedures were in accordance with the Declaration of Helsinki and was approved by the Local Ethical Committee (Protocol n. 2019_0025904).

### 2.2. Architecture Overview

**Figure 1:**
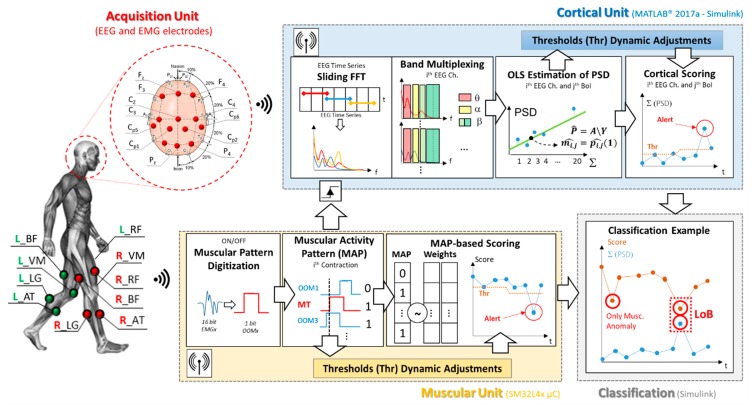
Proposed architecture block diagram. The figure shows the electroencephalography/electromyography (EEG/EMG) experimental setup, as well as the graphical representation of the working flow of each involved block.

Figure 1 shows a block diagram of the proposed loss of balance detection architecture. According to the figure, the system can be divided in four main sections: the acquisition unit, the muscular and cortical units and, finally, the classification block. As depicted in Figure 1, the proposed digital architecture synchronously operates on a STM32L4x microcontroller for the muscular analysis, and by means of Simulink real-time modeling to assess the cortical involvement. The Simulink model has been fully realized by blocks from the Digital Signal Processing (DSP) library in order to be implemented on a microcontroller. 

The system working principle is inspired by our previous works [26,27], which laid the methodological bases for the joint analysis of EEG and EMG signals in the fields of gait analysis and involuntary movements detection. The overall processing chain is detailed in Figure 1.

#### 2.2.1. Acquisition Unit

The acquisition unit consists of a multi-sensing interface that jointly collects data from 10 surface EMGs and an EEG headset. The acquisition equipment has been selected to be fully wireless and wearable, allowing the subject complete freedom of movement.

In more detail, during the test and data collection phases, subjects wore a 32-channels EEG wireless headset (g.Nautilus Research by g.Tec [28]) and a set of 10 wireless surface EMG electrodes (Cometa WavePlus by Cometa srl [29]). According to the experimental measurement setup sketch in Figure 1, thirteen EEG sites have been monitored: F3, Fz, F4, C3, Cz, C4, Cp5, Cp1 Cp2, Cp6, P3, Pz, P4, according to the international 10–20 system. The O2 electrode was used for noise suppression, AFz as ground and the A2 (right earlobe) as the reference electrode. The EEG data were sampled at 500 Hz with 24-bit resolution. 

On the muscular side, 10 surface EMG channels were monitored from following bilateral muscle groups: Anterior tibialis (AT), Lateral gastrocnemius (LG), Vastus medialis (VM), Rectus femoris (RF), and Biceps femoris (BF). The EMG signals were recorded with a sample rate of 2048 Hz and down sampled to 500 Hz (@16-bit resolution) before the transmission.

Data from the 10 EMG nodes are wirelessly streamed to a dedicated gateway, which is mounted on a Nucleo STM32L476RG board via a dedicated Printed Circuit Board (PCB) shield. Then the Muscular Unit algorithm runs on the microcontroller, analyzing the signals sample-by-sample.

Data from the EEG headset are sent to a base station connected via USB to a central computation unit that runs the Simulink model. The base station is also equipped with a 26-pin D-SUB connector used for the parallel reception of 8 digital input pins (DIN). These DINs will be used to receive data and triggers from the microcontroller. On the Simulink model side of the cortical unit, data from the monitored channels are continuously sent to n_ch_ = 13 circular registers, waiting for the enable signal from the muscular block. In this application, the central computation unit that runs the Simulink model consist of a HP Y5L00AE computer embedding an AMD A10-9600P processor (Hewlett-Packard—Palo Alto, CA, USA).

Pre-processing. The EEGs were progressively band-filtered between 1 Hz and 40 Hz by using a built-in 8th order Butterworth filter before the transmission [30]. The EMG node band-pass filters the signal between 15 Hz and 250 Hz before to be sent data to microcontroller [31]. Finally, a numeric notch filter 48–52 Hz has been implemented via the Simulink model for both EEG and EMG signals.

#### 2.2.2. Experimental Protocols

**Figure 2:**
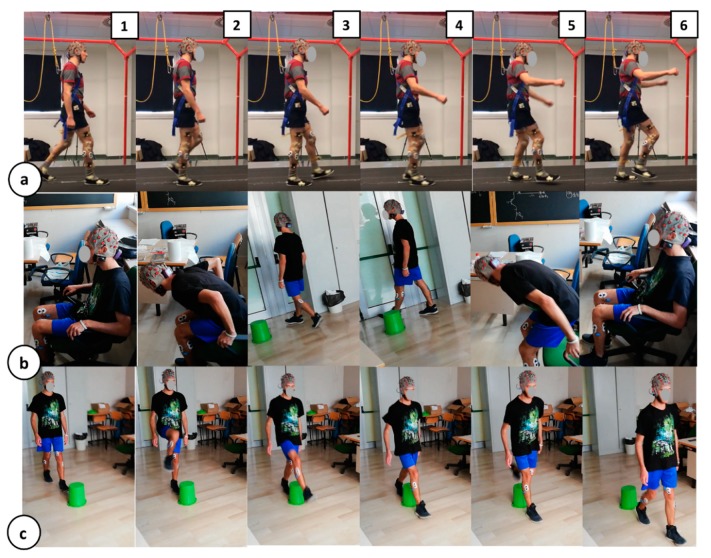
Experimental protocols grid. Each row in the panel represents a 6-frame demo sequence of the experimental protocol carried out by the participants. (**a**) Steady walking to near fall (slip) protocol; (**b**) Chair transfer via timed up and go test; (**c**) Balance-challenging obstacle avoidance.

To test the robustness of the algorithm proposed here and to ensure system suitability for daily-life contexts, the system response was assessed during four different ADL-like tasks. Figure 2 shows, through a snapshot grid, the experimental protocols carried out by the participants. Each row in the figure is composed of 6 frames, realizing a demonstrative sequence of the four experimental tasks:

1. **Steady walking to near fall (slip).** During this protocol, already presented in [26], the participants were asked to manage a slippage, unexpectedly provided during the steady walking by a mechatronic platform, called SENLY [32]. Specifically, the involved subjects underwent a series of 10 consecutive trials where their steady walking was unexpectedly perturbed by a slipping-like perturbation delivered in a pseudo-randomized order. Slippages consisted of a sudden and unexpected movement of one belt toward the antero-posterior (AP) direction. A demo of the protocols is shown in Figure 2a, panels (1) to (6).
2. **Steady walking with sudden curves.** In this protocol, the participants were asked to manage a tight turn around a preset delimiter by keeping the walking speed as constant as possible. The panels (3) and (4) of Figure 2b provides an idea of the protocol described. To evaluate the system specificity against the ADL-like task response, only the contractions related to the sudden curves were collected.
3. **Chair transfer via timed up and go test.** During the TUG test, the participants were asked to stand-up from a chair, walk toward a delimiter, carry out a tight turn around it and go back to the chair to sit- down again. The Figure 2b summarizes in 6 frames the TUG protocol. In this case, the contractions related to the sudden curves are kept in the sudden curves specificity database, while sit-down and stand-up contractions are collected in the dedicated TUG database.
4. **Balance-challenging obstacle avoidance.** This protocol is shown via the 6-frame sequence in Figure 2c. In this protocol, the participants were asked to manage a sequence of obstacle avoidances, by alternating the support foot for every trial. Obstacle avoidance-related contractions have been collected in the dedicated database for the system specificity computation.

#### 2.2.3. ON/OFF Muscular Pattern Extraction

The muscular unit operates on the collected EMGs, generating an ON/OFF binary pattern of muscular activation (OOM—Figure 1) starting from the electrophysiological signal of each monitored muscle. Briefly, the implemented algorithm set OOM = 1 when the muscle is contracted, otherwise reset OOM = 0. This binarization procedure is entrusted to a moving threshold approach detailed in [26,33], because it demonstrated to be able in following the muscle tone changes (e.g., due to fatigue).

The ON/OFF muscular pattern extraction routine implemented on the STM32L4 µC can be briefly summarized by the following steps: the system progressively stores, for each muscle, a time-window containing the last M = 250 samples received.

It then extracts two data blocks: the first one containing the full EEG time-window (M = 250 samples, i.e., ~500 ms) and the second one that includes only the last N = 125 samples (i.e., ~250 ms). The algorithm squares these two vectors, averaging their elements. The resulting EMG power value for the longer time window (PM) acts as adaptive threshold, while the same parameter for the shorter time window (PN) as the instantaneous power. Finally, the two values are compared: if PN > PM, the system set OOM = 1, otherwise OOM = 0. The PM and PN values refresh and progressively adapt to each sample.

This ON/OFF muscular pattern digitization step (Figure 1) generates 10 parallel OOMs (one per muscle), which are sent to the muscular activity pattern (MAP) step according to the block diagram in Figure 1. 

Two OOMs from both the Gastrocnemii are selected to trigger the Cortical Unit. These OOMs will be named master trigger (MT, Figure 1) hereafter. 

To exclude, from the computation, the cortical activity that it is not strictly related to the specific movement, protecting from false alarms in the EEG unit, we selected as MT the gastrocnemius because it uniquely intervenes during the midstance gait phase.

#### 2.2.4. Cortical Involvement Assessment 

Once enabled via MT (side independent), the cortical unit extracts from the circular buffers 13 time-windowed EEGs of 400 samples (~800 ms) preceding the MT onset. 

As a first step, these subset of EEG data undergo the on-line Riemannian artifact subspace reconstruction (rASR) [34]. The rASR is an online/offline artifacts attenuation method for mobile EEG data based on an ASR with Riemannian geometry.

The cortical unit analyzes these artifacts-free brain signals, quantifying the rate of variation in the EEGs power within the five bands of interest identified by authors in [18,19,20,21,22,23]: θ (4–7 Hz), α (8–12 Hz), β I, β II, β III (13–15, 16–20, 21–40 Hz). In more detail, the power spectrum density measurements are done by applying a sliding-window fast Fourier transform (FFT) on the considered EEG subset. For the purpose, the artifacts-free EEG subset is split in 20 overlapped windows long 200-samples with a step of 10 samples, covering the entire length of the subset.

Considering a single EEG window, the application of the FFT leads to a spectral resolution of 2.5 Hz (considering fs_EEG_ = 500 sps and L_win_ = 200 samples), which is suitable for the band multiplexing [26,35].

For each evaluated window, the system extracts a matrix named **S_BoI_** ∈ **R**^nch, nBoI^, with n_ch_ = 13 and nBoI = 5 number of bands involved in the multiplexing.

Each S_BoI_ element is the sum of the spectral contents falling within the selected j-th band due to the multiplexing:

(1) $$S_{\mathrm{BoI}}\left(i,j\right)=\frac{\sum_{k=\left(\mathrm{jth}\;\mathrm{band}\right)}\left(S\left(k\right)\right){|}_{\mathrm{dB}}}{\mathrm{Rg}}\;\;\;\;\;\;\;\;i=1:n_{\mathrm{ch}},\;j=1:n_{\mathrm{BoI}},\;k=1:\mathrm{Rg}$$

where the j-th band can mean the θ (k = 2:3), α (k = 3:5), β I (k = 6:7), β II (k = 8:10), β III (k = 11:16) band range, while Rg is the maximum k index (i.e., length of the j-th band).

The **S_BoI_** is then extended to the 20 overlapped windows, generating a 3D matrix: **Y** ∈ **R**^nch, nBoI, nW^ with nW = 20 number of measurements.

For the sake of clarity, considering a single band of interest (e.g., α band), data from the 20 FFT steps undergo a linear fitting via an ordinary least squares (OLS) estimator according to the equation:

(2) $$\hat{p}\left(i\right){|}_{\alpha }=A\backslash Y\left(i,\alpha ,1\;:\mathrm{nW}\right)$$

where $\hat{p}\left(i\right){|}_{\alpha }$ is the OLS-based parameter vector for the i-th channel on the α band. It contains, in the order, the estimated linear model intercept $\hat{q}=\hat{p}\left(i\right){|}_{\alpha }\left[1\right]$ and the estimated straight-line slope $\hat{m}=\hat{p}\left(i\right){|}_{\alpha }\left[2\right]$. In the same equation (i.e., Equation (2)), **A** is the matrix of the basic functions containing a column of 1 and column of time vector (t = 20:800 ms, step 20 ms). Finally, Y(i,α,1:nW) is the vector that contains the FFT measurements on the i-th channel and the α band. The resulting linear models (OLS estimation—Figure 2) permit to approximate the cortical involvement parameter as the straight-line slope, $\hat{m}$. More details about the EEG computation branch implementation has been provided in our previous works [26,33]. The OLS-based estimation procedure is contextually applied to 13 channels and 5 band of interests, generating 65 $\hat{m}$ values.

#### 2.2.5. Muscular Activity Pattern Extraction

The muscular unit hosted by the microcontroller operates in parallel with the cortical involvement analysis. In this frame, the muscular unit analyzes the 10 parallel OOMs via the MAP extraction routine. This stage aims to analyze the contraction status of each analyzed muscle “in correspondence” of the MT rising edge. Specifically, is time windows of 20 ms (11 samples), 10 ms before and 10 ms after the MT rising edge, is considered. The resulting OOM observation is named **wOOM** ∈ **R**^nEMG, Lw^, where nEMG is the number of monitored EMG nodes and Lw is the number of samples composing the subset. In this application nEMG is 10, while Lw is 11. Also, the element wOOM(i,j) corresponds to the j-th sample of the i-th OOM observation window. In view of this, the **MAP** vector could be mathematically extracted as follows:

(3) $$MAP\left(i\right)=\frac{\sum_{j=1}^{Lw}wOOM\left(i,j\right)}{Lw}=\{\begin{array}{c} \begin{array}{cc} 1 & MAP\left(i\right)>0.5 \end{array}\\ \begin{array}{cc} 0 & \mathrm{otherwise} \end{array} \end{array}$$

According with Equation (3), the outcome of this computation block consists of a 10-element vector (i.e., **MAP**). Each vector element corresponds to a muscle and it is 1 if the considered muscle is active (contracted) for more than half of the observation time, otherwise 0 (i.e., time predominance rule). 

All the MAPs collected during a first brief stage of unperturbed gait or other ADL allow the system to build a first muscular behavior statistic. Specifically, they are used to extract a set of weights. These weights are based on the occurrence of a specific muscle contraction in correspondence of the MT contractions comprising the database. Two weights vectors are derived, one for the right leg (RL) movements and one from the left leg ones (to avoid asymmetry issues). In this way, it is possible to extract the most probable muscular pattern and, thus, a scoring method able to provide a high score if the incoming MAP is similar to the standard pattern, otherwise a low score (in presence of anomaly such as a perturbation). The weights vectors are continuously updated when requested by classification block, according to changes in user rhythms.

#### 2.2.6. Muscular Activity Pattern (MAP)-Based Scoring Section 

In a real-time application context, the MAP-based scoring block (Figure 1) analyzes the incoming MAP binary vector by dot-multiplying it by the related weight vector. For instance, MAP coming from the right Gastrocnemius contraction is dot-multiplied by the right leg-related weight vector and, finally, normalized. The score assignment outcome tends to 1 if the incoming MAP is similar to the muscular standard, otherwise, it tends to 0. 

**Figure 3:**
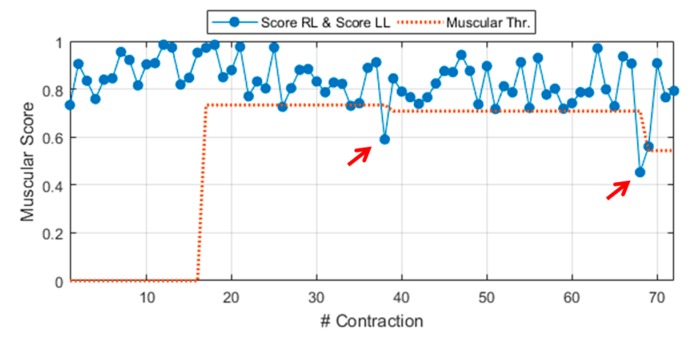
General muscular score (score right leg (RL) ∧ score left leg (LL)) during experimental walking to slip test (Sub. 3 – Trial 4).

Naming **W_R_** ∈ **R**^1, nEMG^ the weight vector from right leg movements, and **MAP_R_** the resulting vector from Equation (3) when the MT is the right Gastrocnemius, the contraction score can be mathematically derived as:

(4) $$\mathrm{Score}\;\mathrm{RL}=\frac{\sum_{i=1}^{\mathrm{nEMG}}MAP_{R}\left(i\right)W_{R}\left(i\right)}{\sum_{i=1}^{\mathrm{nEMG}}W_{R}\left(i\right)}$$

where score RL is the score related to a generic MT contraction from the right leg. Equation (4) can be easily extended to a MT contraction of the left leg MT, by changing the subscript R with L. In this latter case, the score is named score LL (LL, left leg) and it is derived via Equation (4) by considering the proper weight vector **W_L_** and MAPs from the left leg, **MAP_L_**. A demonstrative example of the general muscular score during experimental walking to slip test is shown in Figure 3. The general score includes scores from right leg contractions (score RL) and, also, from left ones (score LL). The figure also shows a preview of the dynamic threshold extracted during the Calibration phase, detailed in Section 2.2.8 (red dotted line).

#### 2.2.7. Cortical Scoring Section

The score assignment embedded in the cortical unit passes through two main steps: the generalization and the lateralization assessment. The generalization step aims to reduce the data to be analyzed (65 vectors of $\hat{m}$ values from 13 channels and 5 bands of interest), providing a qualitative control about the subject’s general cortical involvement. In this respect, the generalization step considers the $\hat{m}$ values on four cortical groups, which roughly identify functional macro areas:

- Supplementary motor area (SMA): F3, Fz, F4;
- Motor area (M1): C3, Cz, C4;
- Sensory-motor area (S1): Cp5, Cp1, Cp2, Cp6;
- Parietal area (PPC): P3, Pz, P4.

This means that, considering an incoming i-th contraction, the system extracts 20 $\hat{m}$ values (one per each functional group extended to 5 bands of interests). To clarify the concept, let us consider the α band involvement on the SMA. The generalized $\hat{m}$ value on the functional group SMA, considering the α band on the i-th contraction can be derived by the following equation:

(5) $${\hat{m}}_{SMA,\alpha }\left(i\right)=({\hat{m}}_{C3,\alpha }\left(i\right)+{\hat{m}}_{C4,\alpha }\left(i\right)+{\hat{m}}_{Cz,\alpha }\left(i\right))$$

The notation can be easily extended to the other formula parameters. 

By contrast with the generalization step, the lateralization one evaluates the incidence of the power increment on a specific side (i.e., left or right) by analyzing the ratio between two specific macro areas: the left side containing {F, C, P}3 and the averaged {Cp1, Cp5}, and the right side that involves {F, C, P}4 and the averaged {Cp2, Cp6}.

The double-check implementation (i.e., generalization and lateralization) is justified by literature findings [19,20,21,22,23], which demonstrated that a reactive response leads to a widespread cortical involvement, while during unperturbed steps or non-challenging ADL, the cortical response remains more lateralized according to the limbs involved in the movement.

The Cortical Scoring block provides 20 values from the generalization step (one per each functional group on 5 bands of interests) and 5 values from the lateralization one (ratio between left and right-side involvement).

#### 2.2.8. Logic Network-Based Classification 

The logic network-based classification block concludes the system workflow according to Figure 1. It consists of two phases: the system adaptive calibration and the logic-network based classifier.

The system adaptive calibration oversees extracting dynamic thresholds (Thr—Figure 1 and Figure 3) for every neuromuscular parameter involved in the classification, i.e., muscular score and the 25 values from the cortical generalization and lateralization steps.

Since the proposed architecture does not embed a learning phase, providing an auto-adaptive turnkey solution, these thresholds are continuously refreshed, contraction by contraction, by means of a sliding observation time window. In this window, the system checks the presence of thresholds lowering via statistical methods (some ADL can drag down the thresholds more than others). If a lowering is recorded, the thresholds are automatically adapted to the next value.

The role of these thresholds is to make the neuromuscular values as handleable as possible, for example, by associating a binary alert to each unexpected behavior. For instance, if the muscular score is below its dedicated threshold (red arrows in Figure 3) the muscular alert goes ON.

In a similar way, the procedure can be applied to the resulting $\hat{m}$ values.

**Figure 4:**
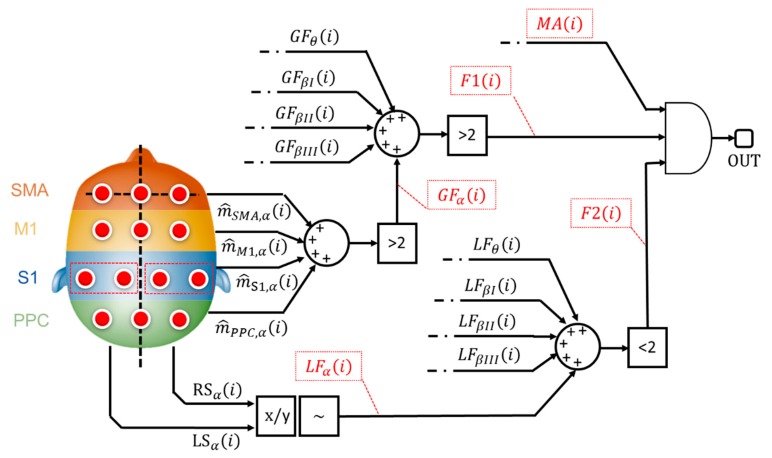
Logic network-based classifier. The flags that contribute to the classification stage are reported in red dotted boxes.

The main goal of the implemented classifier is to cross-relate, among each other, these binary alerts from muscular and cortical sides. Specifically, the classification stage implements a logic network developed on 3 levels as shown by Figure 4.

The 1st level considers the binary alerts from the 4 macro cortical areas (e.g., ${\hat{m}}_{SMA,\alpha }\left(i\right)$- Figure 4)), all over the 5 bands of interest. The system verifies the presence of a widespread increase in brain signal power. If more (>) than 2 cortical areas, for each evaluated band, are involved in the power increment, the architecture sets a generalization flag (GF_α_—Figure 4) to 1, otherwise 0. 

Generalization flags (GFs) from all the evaluated bands are further analyzed. If more (>) than 2 bands are involved in the brain power increase, the 1st level flag, F1 (i) in Figure 4, goes to 1.

The 2nd level analyzes the ratio between the left and the right cortical side (x/y—Figure 4) as described in Section 2.2.7. If the ratio is higher than 1+ε or lower than 1-ε, with ε specific tolerance (~), a lateralized increment is formally recorded. Similarly, the system generates a binary flag, named LF_α_ in Figure 4, which is equals to 1 if a lateralized brain activity is recognized. The system checks the number of lateralization flag as shown in Figure 4: if less (<) than 2 lateralization flags are active, the 2nd-level outcome (F2 (i) in Figure 4) goes to 1.

According to Figure 4, both the 1st-level output (i.e., F1 (i)), from generalization assessment, and the 2nd-level output (i.e., F2 (i)), from lateralization check, are sent to a final AND gate. If both the flags are ON, it means that the system recognized a not-lateralized increment of the cortical involvement. Finally, the classifier runs the 3rd level. This level considers the outcome from the AND gate, toggling the presence of a muscular alert (MA(i)—Figure 4) from the MAP-based scoring block (Figure 1). 

Ultimately, if a not standard muscular behavior, jointly with a widespread and not lateralized cortical behavior, is found, the system classifies the i-th contraction as a potential loss of balance.

The classification output of this logical network can be used to enable a fall-prevention strategy (e.g., through wearable robotics and exoskeletons).

## 3. Results

The proposed system has been validated in near-fall scenarios and ADL-like tasks. During the walking-to-slip test protocol all the participants were secured by a safety harness attached to an overhead as shown in Figure 2a and no falls were reported during the trial. Participants were able to perform multistep recovery reaction to find back their balance. 

Before starting the experimental sessions, all participants signed informed consent. Research procedures were in accordance with the Declaration of Helsinki and were approved by the Local Ethical Committee (Prot. no. 0028266/2019).

Section 3.1 briefly recaps general performance: sensitivity, detection time and specificity concerning steady walking steps versus near fall scenarios. Section 3.2 focuses on the daily life suitability of the system, discussing the method robustness against ADL. Section 3.3 briefly outlines the acquisition equipment features.

### 3.1. Architecture Performance: Loss of Balance versus Steady Walking

As already stated in the state-of-the-art comparison, the performance of a near-fall detection strategy is usually quantified in terms of accuracy and efficiency. According to all the evaluated works [11,12,13,14,15], the accuracy can be evaluated by considering the sensitivity and specificity parameters. Mathematically, the sensitivity can be defined as:

(6) $$\mathrm{Se}\;\left(\%\right)=\left(\#\left(\mathrm{TrNF}\right)/N_{\mathrm{LoB}}\right)\cdot 100$$

where #(TrNF) is the number of correctly detected near fall events (i.e., induced slippages) and $N_{\mathrm{LoB}}$ represents the total number of evaluated loss of balance situations. 

In a complementary way, the specificity is identified as:

(7) $$\mathrm{Sp}\;\left(\%\right)=\left(\#\left(\mathrm{TrADL}\right)/N_{\mathrm{ADL}}\right)\cdot 100$$

where #(TrADL) is the amount of successfully detected ADL-like actions (i.e., walking steps, sudden curves, TUG and obstacle avoidance) and $N_{\mathrm{ADL}}$ is the total number of the evaluated ADL related trials. 

In this section, we analyze results recorded during a real-time application of the walking-to-slip protocol. Two data pools were built: the first dataset is composed of 60 contractions from near-fall scenarios (10 perturbations per 6 subjects), while the second dataset includes 2091 contractions from the steady walking of all the subjects.

The experimentally extracted sensitivity, specificity (related to walking steps) and the detection time values are summarized in Table 2. The proposed multi-sensor architecture shows a sensitivity of 93.33 ± 5.16% and a walking steps vs. loss of balance (slip) specificity of 98.91 ± 0.44%.

The system detection time is about 370.62 ± 60.85 ms, of which—on average—only 21.75 ms are dedicated to the overall computation chain for muscular and cortical units. The computation time comprises: (i) muscle ON/OFF pattern extraction (ii) sliding window FFT, (iii) band multiplexing, (iv) generalization and lateralization step (v) logic network-based classification and (vi) re-calibration of thresholds. Table 2 also shows that in the worst case (i.e., Sub 2, Trial 5) the system demands about 634 ms to intervene, while in the best case (i.e., Sub 6, Trial 4), the system recognizes the loss of balance in about 160 ms.

### 3.2. Architecture Performance: System Robustness against Activities of Daily Life (ADL)

To ensure the daily-life applicability of the proposed architecture, the wearability of the device is not the only constraint. Another important applicability limit lies in the system’s robustness against movements that usually a subject does in his/her domestic environment. These are generally named Activities of Daily Life (ADL). In this respect, this section focuses on the system specificity characterization considering three ADL-like actions as: (i) sudden (and tight) curves, (ii) chair transfers (via TUG test) and (iii) obstacle avoidance.

For the sake of completeness, distinct datasets have been created starting from a real-time application of the system to the three tasks. Each test trial shown in the following consisted of a mixed pattern of these three tasks: walking with sudden curve, TUG and obstacle avoidance. Overall, the offline extraction of specific contractions resulted in a first dataset of 331 contractions (3 subjects) related to tight curves, a second dataset of 512 contractions (3 subjects) from the TUG test and, finally, a dataset that includes 352 contractions (3 subjects) related to the obstacle avoidance.

The steady walking specificity has been evaluated in the previous section by using a dataset of 2091 unperturbed steps (no recovery and near perturbed steps), leading to a value of 98.91 ± 0.44% that we assume as a final characterization parameter for the sake of readability.

**Figure 5:**
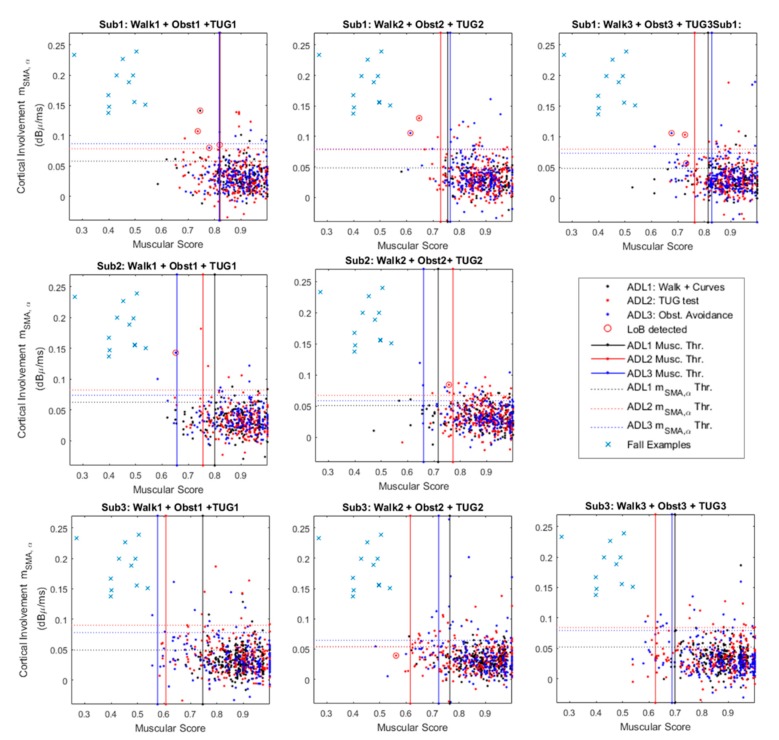
Cortico-muscular involvement planes for the 3 analyzed subjects. The figure merges contractions from three different Activities of Daily Life (ADL): curves (black), timed up and go (TUG) test (red) and obstacle avoidance (blue). The panels show the false alarms (red circles) and a comparison group (blue crosses) that represents the typical values occurring during a fall-related master trigger (MT) contraction.

Figure 5 provides a graphical characterization of the system robustness against the ADL.

The figure shows three panels per subject, except for Sub. 2 that did not perform the obstacle avoidance during the third test trial. Each single panel shows a 2D plane in which the x-axis reports the muscular score (MS in the following) as defined in Section 2.2.3, while the y-axis refers to the ${\hat{m}}_{SMA,\alpha }$ values, obtained according to Equation (5). Each point on the plane has coordinates {MS(i), ${\hat{m}}_{SMA,\alpha }\left(i\right)$} where “i” is the i-th MT contraction that led to features extraction. Each single point identifies two features that contributes to the final classification of the specific contraction.

The panel also shows 3 thresholds that are constant along the x-axis (solid red, blue and black lines). These thresholds are those that operate on the muscular score (i.e., MS), acting as shown in Figure 5 by considering the median on an observation window of 15 consequent contractions.

This means that the system slowly adapts to the worst thresholds among the evaluated ones.

For example, considering The Sub.1–Test: 2, the worst MS-related threshold is linked to the ADL3: obstacle avoidance (Figure 5). It means that during its steady functioning, the system will recognize as a real dangerous situation only MS below the obstacle avoidance related threshold (i.e., ADL3 Musc. Thr.—Figure 5).

The panels also provide y-axis constant thresholds that refers to virtual upper limits for ${\hat{m}}_{SMA,\alpha }$. These thresholds act in a similar manner of the previously presented ones. Considering for example data from Sub.3–Trial 2 in Figure 5, this means that during its steady functioning the system will recognize a dangerous situation only for ${\hat{m}}_{SMA,\alpha }$ above the obstacle avoidance threshold (i.e., ADL3 ${\hat{m}}_{SMA,\alpha }$ Thr.—Figure 5). Ultimately, real dangerous situations for both muscular and cortical involvements lie in the top-left rectangles delimited by the leftmost MS-related threshold and the highest ${\hat{m}}_{SMA,\alpha }$-related threshold. On these panels, some contractions wrongly recognized as losses of balance highlighted via red circles. In this context, we must consider it as incorrect classification, reducing the specificity as per Equation (5).

Finally, for the sake of comparison, Figure 5 reports as blue crosses some slip-related coordinates {MS (i), ${\hat{m}}_{SMA,\alpha }\left(i\right)$}. These coordinates have been extracted from the walking versus slip dataset (Section 3.1) considering two points per each analyzed subject. This shows how the two groups ADL1,2,3 and slips could be easily divided in clusters.

To give a complete overview of the system robustness against ADL, Table 3 summarizes the experimental results in terms of specificity from each protocol carried out (ADL 1, 2 and 3 Sp. (%)), as well the single test (Tasks 1, 2, 3 Sp. (%)) and subject-related (Sub. Sp. (%)) specificities. Analyzing data in Table 3 and in Figure 5 it is possible to state that, overall, the system showed a specificity of 98.91 ± 0.44% in steady walking steps’ recognition (see Table 2), 99.62 ± 0.66% in sudden curves successfully detection, 98.95 ± 1.27% of correct recognition in contractions related to TUG tests. Finally, during the balance-challenging obstacle avoidance protocol the specificity reached 98.43 ± 0.88%.

In order to provide a comparison with the state-of-the-art solutions, already presented in the introduction, Table 4 summarizes some specific features for each analyzed work. In particular, Table 4 focuses on recognized classes, the system performance in terms of accuracy and efficiency and the applicability tabs. Data from Table 4 show how the proposed system ensures very competitive specificity value (i.e., 98.9%).

### 3.3. Acquisition Equipment Features

Once the algorithm robustness against ADL recognition is verified, the system’s applicability to ordinary life imposes another constraint: wearability. The chosen equipment should address the wearability constraints, which according to [36] can be summarized briefly in three macro-categories: (i) encumbrance (ii) biomechanical effects and (iii) comfort.

Considering the former constraint, the physical dimensions of the wearable will be paramount. These dimensions include the size, weight and the distribution of the weight of the wearable on the body.

Secondly, the functional placement of the sensor nodes may affect the posture and musculoskeletal loading of the wearer. Finally, the sensors’ node placement must avoid discomfort, favoring regular movements (e.g., walking or sitting) and non-biased postures.

To continuously analyze and characterize the subjects’ cortical and muscular dynamics in several different ordinary life scenarios, the here-proposed experimental setup consists of a 32-channel wireless EEG headset (g.Nautilus Research) by g.Tec [28] and 10 wireless surface EMG nodes (Cometa Wave Plus) by Cometa Systems srl [29]. Table 5 provides information about the acquisition equipment comprising the set-up. For each device, the table reports the number of monitored nodes or channels, equipment features such as the size and weight, as well as the electrode characteristics and device parameters: wireless transmission range and protocol, resolution and sampling frequency. Table 5 demonstrates how the equipment choice ensures a fully wireless and low-encumbrance solution, validating the applicability in an indoor monitoring scenario. Despite this, the use of gel-based or pre-gelled electrodes could be considered uncomfortable for long-time acquisition. In this respect, the system can be considered reliable for 4 hours’ acquisition, before the need to refill the gel to ensure the right input impedance to the amplifier.

## 4. Discussion

The detection of near falls is an emerging area of research that is contextually growing with the development of an increasing number of miniaturized and power-efficient wearable devices [7]. Supported by accumulated evidence on fall detection [7,8,9,10,11,12,13,14,15,16,17,18,19,20,21,22,23], the clinical utility of this investigation involves the unobtrusive and continuous monitoring of activities of daily life in populations at high risk of falling. This kind of strategy (i.e., near fall detection) can be useful to identify issues to be further addressed to prevent falls, associated injuries or simply improve the efficiency of already existing pre-impact fall-detection architectures.

In this study, we have proposed a novel wearable architecture that exploits electrophysiological signals from brain and lower limb muscles to discriminate a near-fall scenario (i.e., unexpected slippages) from an activities of daily life. The proposed system realizes a turnkey solution, which can adapt its function to the user neuromuscular rhythms, without any long and fatiguing learning stage.

Results in Section 3.1 showed how the proposed architecture demands about 370.62 ± 60.85 ms to carry out a binary classification (i.e., ADL vs. near fall). As stated in the same section, the overall computation chain of muscular and cortical units requires, on average, 22 ms to be completed.

The remaining time, i.e., ~350 ms, with its high variability (see Table 2), is related to the muscle that has been selected as a master trigger. In fact, it should be reminded that the system starts working from the contraction onset of the gastrocnemius (right or left independently).

The times related to this physiological process remain hard to determine with certainty. In this respect, the response times of the gastrocnemius constitute unavoidable delays in recognizing losses of balance and largely determine the efficiency of the system. Further investigation should be conducted in order to find another muscle bundle that can: (i) uniquely define a gait phase, (ii) activate itself faster in a perturbation context, (iii) ensure repeatability during the contraction timing when the near-fall scenario occurs.

The detection times achieved are competitive with respect to the state-of-the-art solutions, highlighting the system applicability in contexts of postural recovery strategies implementation [17].

Concerning the results in Section 3.2 that analyze the system robustness against the ADL, an interesting evaluation should be undertaken into the losses of balance detected below the worst thresholds, such as Sub1-Test1 and Sub2-Test2 in Figure 5. In these cases, offline checks verified that the threshold was slowly adapting to the final value, causing transitional “false alarm” (wrongly loss of balance detection). Within this, the threshold adapting procedure should be improved and speeded up, while keeping high sensitivity and specificity.

Another noteworthy case is that shown in Sub3- Test 2 (Figure 5). In that case, it seems that a loss of balance is detected below the cortical thresholds. It is important to remember that these panels show only the ${\hat{m}}_{SMA,\alpha }$ values, nevertheless, we must consider that the analyzed problem is hyper-dimensional, because we should take into account other four bands of interest and the remaining 3 cortical groups.

Moreover, in Figure 5 it is notable that the Sub. 1 experienced a high number of false alarms with respect to the following two subjects. This result could be related to the protocol improvement asked on-going to the last two participants. This improvement mainly concerns the sit-down and stand-up movements during the TUG. In fact, since the rejection algorithm rASR has not been optimized to reject the muscular artifacts from strong contractions of the deltoids, the EEG acquisitions were spoiled by unpredicted artifacts. In this respect, further investigations are still ongoing aiming to extend the range of applicability of the implemented rASR algorithm.

## 5. Conclusions

In this paper, we proposed and validated a novel architecture for the losses of balance recognition. The proposed system, optimized for unexpected slippages, addressed some still open challenges related to the daily life applicability of this kind of system. Design and verification constraints mainly concern the need for high specificity and system robustness against ADL. In this respect, the proposed algorithm has been tested on five different tasks: sudden curves, chair transfers via the timed up and go test, balance-challenging obstacle avoidance and, of course slip-induced loss of balance. To ensure the ordinary life suitability, the proposed architecture has been fully based on wearable and wireless acquisition devices. Specifically, the architecture exploits electrophysiological measurements from 10 EMG electrodes and 13 EEG channels. The collected data are analyzed by the muscular unit, hosted by a STM32L4 microcontroller, and the cortical unit, which is implemented on a central computation unit via Simulink modeling. The first realizes a binary ON/OFF pattern from muscular activity (10 EMGs) and triggers the cortical unit that evaluates the contraction-related cortical involvements in terms of EEG responsiveness.

This parameter is evaluated as the variation behavior in the EEG PSD, considering five bands of interest. The neuromuscular features from both the computation units are sent to a clinical evidence based logical network. It embeds a set of automatically adaptive thresholds, which follow the user rhythms. Experimental validation on 9 healthy subjects showed that the system could react in a time compliant with fall-detection architectures constraints (i.e., 370.62 ± 60.85 ms). It also ensures a fall detection sensitivity of the 93.33 ± 5.16%. During the ADL tests the system showed a specificity of 98.91 ± 0.44% in steady walking steps’ recognition, 99.61 ± 0.66% in successful sudden curves detection, and 98.95 ± 1.27% of correct recognition in contractions related to TUG tests. Finally, during the balance-challenging obstacle avoidance protocol the specificity reached the 98.42 ± 0.90%.

These preliminary results show promising accuracy values that, jointly with the system wearability (wireless acquisition devices), make the system potentially suitable for daily life application. Moreover, the achieved detection time (i.e., ~371 ms) is conservatively below 550 ms, which is considered as the maximum intervention limit for the implementation of countermeasures aimed at restoring the balance of the subject [17]. It ensures the system applicability to improving a fall-detection strategy.

Some drawbacks that need future larger and higher quality studies concern the acquisition devices and the generalization of the implemented method. In fact, the use of wireless sensors (EEG/EMG) theoretically ensures the system’s wearability. Nevertheless, future perspectives concern the study of more comfortable solutions able to provide the same electrophysiological patterns, e.g., by using textile-based sensors arrays. The second weak point under investigation is the muscle to be selected as the master trigger to provide a quasi-deterministic delay, improving the system efficiency as a logical consequence.

## Figures and Tables

**Table 1:** State-of-the-art overview: features, performance (accuracy and efficiency) and applicability.

Reference		[11]	[12]	[13]	[14]	[15]
**Acquisition Devices ^1^**		n.7 IMU 6DoF sensor (GYR + ACC)	MCS: total body	MCS: total body	n.1 IMU 6DoF sensor (GYR + ACC) on waist (close CoG)	Hips Encoder on APO + MCS: validation on lower limbs
**Fall Indicators ^2^**		Means and variances of X, Y, Z for each ACC and GYR	Acceleration and VV of upper arms, trunk, tibia and head	Acceleration of all the monitored body segments	VV by integrating ACC data	ErF between Current Hips angle and predicted one
**Classification Approach ^3^**		**ML:** RBF kernel SVM	**Multiple Thr + Statistics:** threshold based on an ARIMA model	**ML:** (1) accelerations analysis by ICA (2) ANN	**Single Thr:** user-specific pre-impact VV threshold	**Single Thr:** increment of the ErF
**Recognized Classes ^4^**	**ADL**	Walking, Standing, correct chair transition, lying, picking up objects, ascending and descending stairs	Walking, Standing	Walking, Standing	Walking, Standing, correct chair transition, lying, picking up objects, ascending and descending stairs	Walking, Standing
	**Near Fall Scenarios**	Slip, Trip, Incorrect chair transfer, misstep (*recovery*)	Slip (*recovery*)	Slip (*recovery*)	Slip, Trip, Incorrect chair transfer, misstep (*recovery*)	Slip (*recovery*)
**System Performance**	**Se (%)**	80–96	88.5	92.7	95.2	92.7
	**Sp (%)**	90.8–99.2	92.9	98.0	97.6	98.0
	**DT (ms)**	offline	Mean: 680.00	Mean: 351.00	Mean: 469.00	Mean: 403.0
**Applicability ^5^**	**OL**	✘	✘	✘	✔	✘
	**Clin.**	✔	✔	✔	✔	✔
	**FD**	✘	✘	✔	✔	✔

^1^**Acquisition Devices Acronyms:** IMU: inertial measurement unit, DoF: degree of freedom, GYR: gyroscope, ACC: accelerometer, MCS: motion capture system, CoG: center of gravity, APO: Active Pelvis Orthosis. ^2^ **Fall Indicators Acronyms:** VV: vertical velocity, ErF: error function. ^3^ **Classification Acronyms:** ML: machine learning, RBF: Radial Basis Function, Thr: threshold, SVM: support vector machine, ARIMA: autoregressive integrated moving average, ICA: independent component analysis, AR: Autoregression. ^4^ **Classes Acronyms:** ADL: Activities of Daily Life ^5^ **Applicability Acronyms:** OL: ordinary (or daily) life applicability, Clin.: Ambulatory applicability, FD: Fall-detection system suitability.

**Table 2:** Proposed architecture accuracy and efficiency: near falls vs. steady walking.

Subject	Se (%)	SpWS ^1^(%)	DT (ms)	
			µ±σ	Max|Min
**1**	90.00 (9/10)	99.22 (386/389)	369.83 ± 97.49	536.11 | 202.02
**2**	100.00 (10/10)	98.32 (292/297)	436.72 ± 86.66	634.21 | 371.15
**3**	90.00 (9/10)	98.71 (308/312)	299.76 ± 107.99	432.00 | 194.60
**4**	90.00 (9/10)	98.55 (339/344)	355.85 ± 151.38	581.35 | 198.73
**5**	90.00 (9/10)	99.46 (370/372)	446.72 ± 112.89	626.45 | 374.36
**6**	100.00 (10/10)	99.20 (374/377)	314.82 ± 105.34	501.23 | 160.42
**Avg ^2^**	**93.33 ± 5.16**	**98.91 ± 0.44**	**370.62 ± 60.85**	**634.21 |160.42 ^3^**

^1^**SpWS**: specificity strictly related to walking steps as not loss of balance actions; ^2^**Avg**: averages among all the analyzed subjects (Sub. 1-6); ^3^ Values refer to the highest maximum and lowest minimum values among the reported data.

**Table 3:** ADL-related specificity extraction.

**Curves**	**Task 1 Sp. (%)**	**Task 2 Sp. (%)**	**Task 3 Sp. (%)**	**Subject Sp. (%)**	**ADL1 Sp. (%)**
Sub. 1	96.55 (28/29)	100.00 (23/23)	100.00 (26/26)	98.85 ± 1.99	99.62 ± 0.66
Sub. 2	100.00 (34/34)	100.00 (36/36)	100.00 (35/35)	100.00	
Sub. 3	100.00 (51/51)	100.00 (48/48)	100.00 (49/49)	100.00	
**TUG**	**Task 1 Sp. (%)**	**Task 2 Sp. (%)**	**Task 3 Sp. (%)**	**Subject Sp. (%)**	**ADL2 Sp. (%)**
Sub. 1	97.50 (39/40)	98.21 (55/56)	96.88 (62/64)	97.52 ± 0.67	98.95 ± 1.27
Sub. 2	97.91 (47/48)	100.00 (56/56)	100.00 (48/48)	99.30 ± 1.20	
Sub. 3	100.00 (64/64)	100.00 (64/64)	100.00 (72/72)	100.00	
**Obst. Avoidance**	**Task 1 Sp. (%)**	**Task 2 Sp. (%)**	**Task 3 Sp. (%)**	**Subject Sp. (%)**	**ADL3 Sp. (%)**
Sub. 1	95.24 (40/42)	98.61 (71/72)	98.44 (63/64)	97.48 ± 1.89	98.43 ± 0.88
Sub. 2	100.00 (30/30)	97.22 (35/36)	-	98.61 ± 1.96	
Sub. 3	100.00 (30/30)	97.61 (41/42)	100.00 (36/36)	99.21 ± 1.37	

**Table 4:** Comparison of proposed architecture’s performance.

Reference	Recognized Classes		System Performance			Applicability		
	ADL	Near Falls	Se (%)	Sp. (%)	DT (ms)	OL	Clin.	FD
**[11]**	Walking, Standing, correct chair transition, lying, picking up objects, ascending and descending stairs	Slip, trip, incorrect chair transfer, misstep (*recovery*)	80.0–96.0	90.8 – 99.2	offline	✘	✔	✘
**[12]**	Walking, Standing	Slips (*recovery*)	88.5	92.9	680.00	✘	✔	✘
**[13]**	Walking, Standing	Slips (*recovery*)	92.7	98.0	351.00	✔	✔	✔
**[14]**	Walking, Standing, correct chair transition, lying, picking up objects, ascending and descending stairs	Slip, trip, incorrect chair transfer, misstep (*recovery*)	95.2	97.6	469.00	✔	✔	✔
**[15]**	Walking, Standing	Slip (*recovery*)	97.6	98.0	403.00	✘	✔	✔
**This Work**	Walking, Standing, Sudden curves, Chair transitions (TUG), Obstacle avoidance	Slips (*recovery*)	**93.3**	**98.9 ^1^**	**370.62**	✔	✔	✔

^1^ The Sp(%) value has been evaluated as the average between the four specificity values (i.e., steady walking, curves, TUG, obstacle).

**Table 5:** EEG/EMG acquisition devices features.

Signal	Num.	Equipment Features	Electrode		Transmission Range	Resolution (Sampling FrEquation)
			Size (mm)	Type		
**EEG**	13 channels	**EEG Headset:** **Back Head Station:** 70 × 55 × 30 mm Weight: 145 g **Headset:** Full-scalp elastic cap Weight: 12 g *Wireless* 10 h continuous acquisition @ 500Hz	16 × 10 × 5	Active Gel based Sintered Ag/AgCl probe	**Modulo RF:** XVV-MEGA22M00 (IEEE 802.15.4 WPAN @ 2.4GHz) **Indoor Range:** 17 m with 2.3–2.9dBm	24 bit (@500 Hz)
**EMG**	10 nodes	**EMG Single Node:** 33 × 23 × 19 mm Weight: 12 g Wireless 12 h continuous acquisition @ 2048 Hz	18 × 12 × 5	Active Pre-Gelled Sintered Ag/AgCl holder ring	**Private protocol:** Y9SMPTX 2.402–2.48 GHz **Indoor Range:** 15 m (+3dBm)	16 bit (@2048 Hz ↓ 500 Hz)
